# Comparative immunohistochemical characterisation of a teratoma in a domestic duck (*Anas platyrhynchos*) and a teratocarcinoma in a muscovy duck (*Cairina moschata*)

**DOI:** 10.1186/s13028-025-00791-z

**Published:** 2025-04-11

**Authors:** Deborah Johanna Eikelberg, Melanie Stoff, Martin Peters, Marko Legler, Ingo Gerhauser, Peter Wohlsein

**Affiliations:** 1 Department of Pathology, University of Veterinary Medicine, Bünteweg 17, D-30559 Hannover, Germany; 2 Chemisches und Veterinäruntersuchungsamt Westfalen, Zur Taubeneiche 10-12, D-59821 Arnsberg, Germany; 3 Clinic for small mammals, reptiles and birds, University of Veterinary Medicine, Bünteweg 9, D-30559 Hannover, Germany

**Keywords:** Coelomic teratoma, Coelomic teratocarcinoma, Duck, Histology, Immunohistochemistry

## Abstract

**Background:**

Neoplasms consisting of two or more germinal layers are classified as teratomas if benign or as teratocarcinomas if malignant. Teratomas are rare tumours occurring in a wide range of species including mammals, birds and reptiles and only a few cases have been reported in ducks with infrequent documentation of immunohistochemical characterisation. Teratocarcinomas occur rarely and, to the authors’ knowledge, have not been described or immunohistochemically characterised in ducks, yet. Therefore, the clinical and pathological presentation of a teratoma in a mulard duck and a teratocarcinoma in a muscovy duck are described. In addition to a histologic examination, both tumours were characterised by applying a panel of immunohistochemical markers previously tested on duck tissue.

**Case presentation:**

A mulard duck (*Anas platyrhynchos x Cairina moschata*) showed a mass in the cranial coelomic cavity histologically diagnosed as tridermic teratoma. A caudal coelomic mass in a Muscovy duck (*Cairina moschata*) was histologically diagnosed as a teratocarcinoma with metastases to the liver, mesentery and intestinal wall. An extensive immunohistochemical examination for a detailed characterisation and comparison with duck control tissue was performed in both cases, highlighting various components of the neoplastic tissues including lymphocytes, nervous and endodermal components.

**Conclusions:**

To the authors’ knowledge, this is the first report of a teratocarcinoma in a duck with intense immunohistochemical characterisation and comparison with a teratoma in another duck. Immunohistochemistry enables a more profound examination of the histogenetic composition of such neoplasms compared to histology alone. Both neoplasms should be considered as differentials for masses in body cavities of ducks. During these examinations, a large spectrum of antibodies specific for different tissues and cells were tested on duck control tissue which can be of help for immunohistochemical examinations on avian tissues in the future.

**Supplementary Information:**

The online version contains supplementary material available at 10.1186/s13028-025-00791-z.

## Background

Neoplasms consisting of two or more germinal layers, i.e. ectoderm, neuroectoderm, mesoderm and endoderm [1, 2], are classified as teratomas if they are benign or as teratocarcinomas if they are malignant. Teratomas are rare tumours occurring in a wide range of species including mammals, birds and reptiles [1, 3–6]. Anatomically, gonadal teratomas derived from germ cells are distinguished from extragonadal teratomas, in which embryonic stem cells are assumed to be the cells of origin [1, 3, 7–9]. The individual germinal layers can exhibit variable degrees of differentiation, resulting in a complex composition of teratomas. Morphologically, teratomas as well as teratocarcinomas of mammalian species appear as solid masses with eventually grossly visible epithelial cysts, hair, teeth, cartilage and bone embedded in soft tissues. Histologically, epidermis, hair follicles in mammals, intestinal, respiratory or renal epithelium, neural, fibrous, and fatty tissue, as well as musculature are found [3]. In birds, instead of hair follicles, the presence of feather follicles [10, 11] and feathers [12] is described. Classification of benign teratomas and malignant teratocarcinomas is based on histological features and includes the degree of germinal layer differentiation (i.e. well-differentiated mature tissue components vs. poorly differentiated, immature embryonic stem cells), presence of local invasion and formation of metastases [1, 13–15]. Both, teratomas and teratocarcinomas, have been described in different avian species. Teratomas, for example, have been documented for chicken [4, 10, 16–20], ducks [20–27], black headed gull [28], domestic goose [20, 29], domestic turkey [30], fantail pigeon [31], red-crowned Amazon Parrot [32] and great blue heron [12]. Teratomas in birds often originate in the gonads and in chicken with a higher prevalence in the testis than in the ovary [4, 16, 20–22, 27]. Other locations include the cranial coelomic cavity [24], or intracranial [26, 31], periocular [30], subcutaneous [32] and retrobulbar localisations [12, 19], as well as in multiple organs [28]. In some cases, the exact origin remained unknown [23, 27, 29]. In ducks, teratomas were localised in the coelomic cavity [21, 23–25, 27, 33], subcutis [26] as well as in gonads [21]. Teratomas affect both, males and females [11, 12, 23, 24, 27, 29–31]. In birds, there is little knowledge about the etiology of teratomas. Besides spontaneous development, teratomas might be caused by viral infections [34] or intoxication with zinc [16].

For teratocarcinomas in avian species, only single cases have been described such as an ovarian teratocarcinoma and a teratocarcinoma of unknown origin in emus [35, 36] or a malignant retrobulbar and intracranial teratoma in lesser kestrel [37].

Besides histological examination of teratomas and teratocarcinomas, immunohistochemical characterisation of these tumours has been performed rarely in avian species [27, 30, 31, 35, 37]. This case report describes clinical and histomorphological features of a teratoma and a teratocarcinoma in two ducks and comparative comprehensive immunohistochemical properties. Applied antibodies reactive with various cytological marker molecules in epithelial, mesenchymal, nervous and neuroendocrine tissues were tested on duck control tissue including respective organs with commonly immunoreactive structures (Additional file 1; Additional file 2).

## Case presentation

**Fig. 1:**
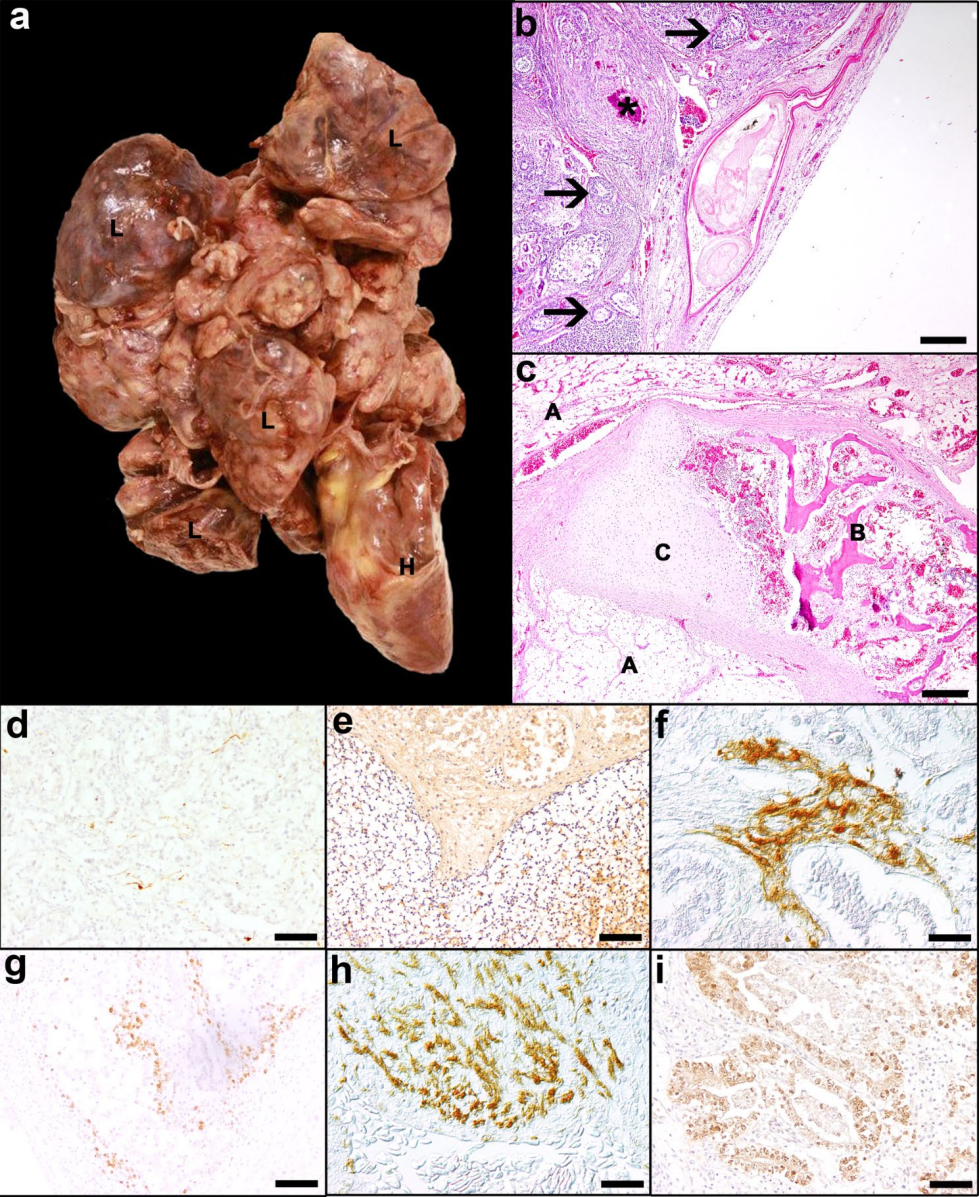
Mulard duck (case 1) with a teratoma in the cranial coelomic cavity. **a** Coelomic mass (11 × 13 × 3.5 cm) attached to the heart (H) and lung (L). **b** Multiple epithelial cysts containing desquamated, orthokeratotic epithelial cells, blood-filled cysts (asterisk) and glandular structures (arrows), H&E. Bar: 100 μm. **c** Islands of trabecular bone tissue (B) containing haematopoietically active bone marrow as well as cartilage clusters (C) surrounded by adipose tissue (A), H&E. Bar: 200 μm. **d** Pan-Neurofilament (NF) positive axonal structures. **e** Multifocal weakly Olig2 positive cells in a tissue with a honeycomb-like appearance. **f** Glial fibrillary acidic protein (GFAP) positive astrocytes. **g** CD3 positive T-lymphocytes. **h** Desmin expression in a bundle of spindeloid cells. **i** Sox2 positive, columnar to cuboidal cells in tubular structures. **d** - **i**: Bars: 50 μm

A 2.5-month-old, female mulard duck (*Anas platyrhynchos x Cairina moschata*; case 1) showed respiratory signs after a flight and died spontaneously. Previous to the respiratory distress, the bird was clinically normal. Full necropsy was performed and revealed an 11 × 13 × 3.5 cm large, well-demarcated, partly encapsulated, multilobulated, beige-coloured mass in the cranial coelomic cavity. The mass was composed of soft and firm tissues with small cysts attached to the serosa of the pericardial sac, the lung and the base of the heart compressing the liver and the lung (Fig. 1a). The cut surface of the mass revealed mostly yellowish-white components with multifocal, cystic structures filled with gelatinous, brownish fluid. The gonads were small and without significant findings, interpreted as juvenile. Samples of all organs and the coelomic mass were collected and fixed in 10% neutral buffered formalin. Tissue samples were routinely embedded in paraffin wax and 4 μm thick sections were stained with haematoxylin and eosin (H&E). Moreover, due to the medical history of respiratory signs, avian influenza virus infection was excluded applying polymerase chain reaction.

Histologically, the mass was composed of tissues from all primordial germ layers including ectoderm, neuroectoderm, mesoderm and endoderm. Ectodermal components included cystic structures lined by well differentiated squamous epithelium and accumulated orthokeratotic keratin lamellae (Fig. 1b). Neurectodermal components were present as neurons and small oligodendrocyte-resembling cells arranged in a honeycomb pattern. The mesodermal parts of the tumour consisted of islands of hyaline cartilage and irregularly arranged striated muscle cells, adipose tissue, chondrocytes, bone with bone marrow and haematopoiesis (Fig. 1c). Connective tissue was present in the neoplastic mass either as supporting stroma, e.g. for epithelial structures, but also as island-like areas, e.g. adjacent to cartilaginous or osseous components, which were regarded as neoplastic. The presence of more or less uniform distributed blood vessels was interpreted as stromal vascularization, whereas clustered blood vessels of varying diameter were considered as neoplastic proliferation. Endodermal components were characterised by tubulo-adenoid structures with single- or multilayered columnar epithelium. Mitotic figures were rarely found in all cell types from the three germinal layers (on average 1 mitotic figure in a field area of 2.37 mm²).

**Fig. 2:**
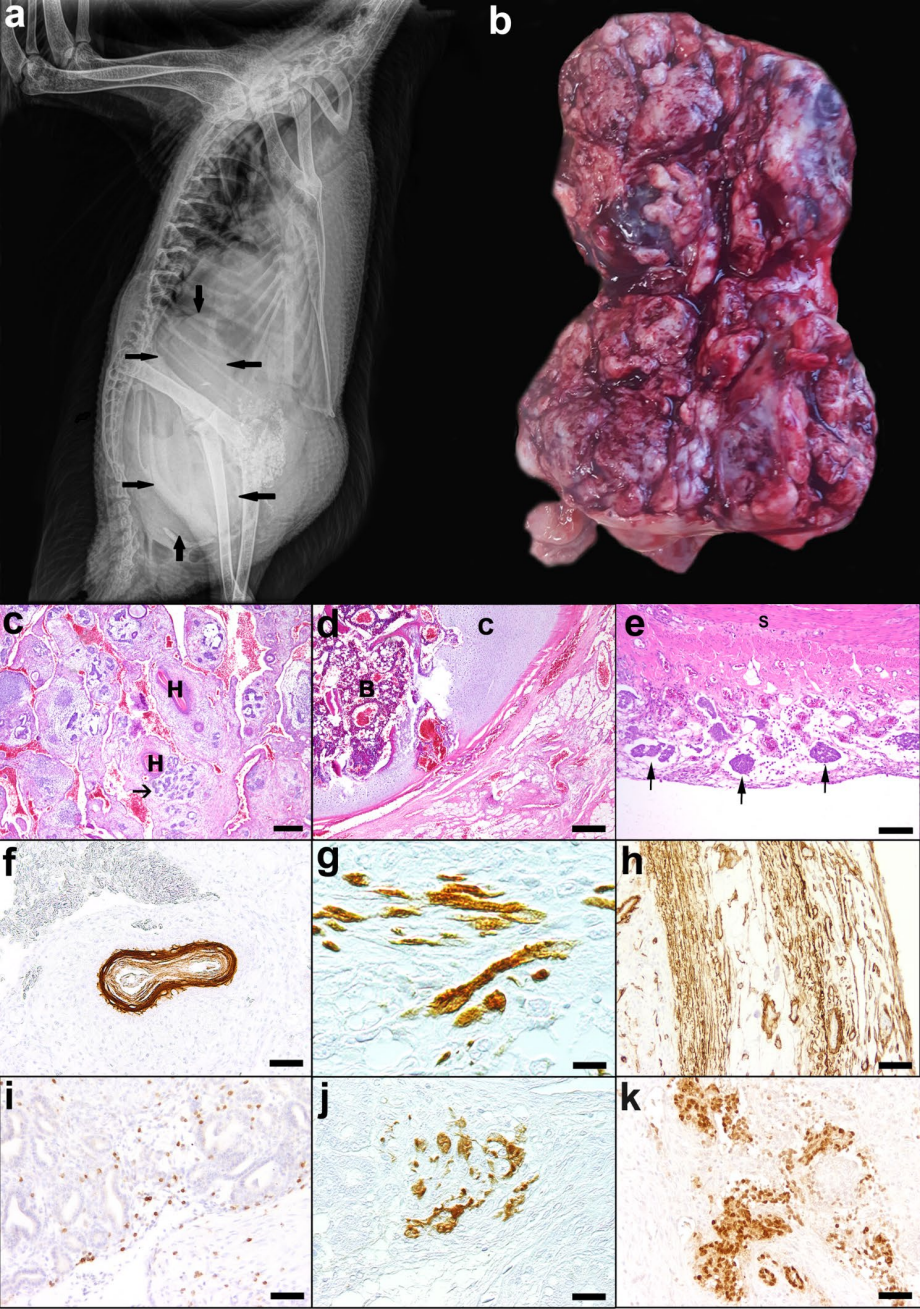
Muscovy duck (case 2) with a metastasising teratocarcinoma. **a** X-ray of the duck with a coelomic mass (arrows) compressing internal organs. **b** Coelomic mass (10 × 12 × 3 cm) with coarse nodular surface. **c** Epithelial proliferation with squamous differentiation and keratin pearls (H) and epithelial nests (arrows). H&E. Bar: 200 μm. **d** Formation of cartilage (C) and bone tissue (B). H&E. Bar: 200 μm. **e** Small intestine with numerous embolised tumour cell aggregates in ectatic lymphatics of the serosa (*lymphangiosis carcinomatosa*); S = smooth muscle layer of the intestine; H&E, Bar:100 μm **f** Cytokeratin (CK) 20 positive keratin pearl. **g** Desmin expressing spindeloid cells with partially visible cross-striation. **h** Smooth muscle actin (SMA) positive fibers. **i** CD3 positive T-lymphocytes. **j** Glial fibrillary acidic protein (GFAP) positive astrocytes **k** Sox2 positive, columnar to cuboidal cells in tubular structures. **f** - **k**: Bars: 50 μm

A three-year-old, female Muscovy duck (*Cairina moschata*; case 2) had normally laid eggs in 2019, which stopped in 2020. The duck developed apathy and egg binding was suspected by the owner. Upon clinical examination diarrhoea and a distended abdomen were noticed. X-ray revealed a mass in the area of the oviduct (Fig. 2a). On laparotomy, multiple masses in the abdomen were present, which involved ovaries, coelomic serosal surfaces, liver, and small intestine. Due to the multi-organ involvement the animal was euthanised. Parts of the mass, liver, and small intestine were fixed in 10% neutral buffered formalin and submitted for histological examination, as the owner refused a complete necropsy of the animal. Tissue samples were routinely embedded in paraffin wax.

The submitted, 10 × 12 × 3 cm large part of the mass was poorly demarcated, multilobulated, beige-coloured and consisted of several nodules (Fig. 2b). Nodules were composed of firm and soft areas infiltrating into the mesentery and peritoneal serosa, also compressing and infiltrating the liver, the wall of the small intestine and the ovaries. The cut surface showed a beige-coloured to greyish, multifocally red, multilobulated, mostly solid, partly cystic appearance. Moreover, the mesentery attached to the liver was thickened. Because the complete carcass was not accessible for necropsy, the gonads were not examined.

Histologically, the neoplasm of case 2 was diagnosed as an infiltrative and metastasising teratocarcinoma. The tumour was composed of ectodermal components with squamous epithelium and keratin pearls (Fig. 2c), mesodermal components including large amounts of fibrous tissue, clustered vascular proliferations, cartilage and bone with bone marrow (Fig. 2d) and endodermal components with adenoid structures composed of cuboidal to columnar cells. Isoprismatic, medium sized epithelial cells were arranged in nests and tubules displaying mild to moderate anisocytosis and anisokaryosis. They contained large nuclei with chromatin displaced to the nuclear margin, and one or two distinct, small to medium sized, basophilic nucleoli. This cell population showed up to 14 mitotic figures in a field area of 2.37 mm². Additionally, solitary or aggregated tumour cells were present in ectatic lymphatics of the small intestinal serosa (Fig. 2e). These masses contained not only the above described epithelial cells, but also large amounts of fibrous tissue. In the mass attached to the liver, squamous epithelium with formation of keratin pearls was also present. Adjacent to the mass a granulomatous inflammation with multinucleated giant cells was observed. There was no evidence of fungal structures applying the Periodic acid-Schiff (PAS) reaction and Grocott´s methenamine silver impregnation. Moreover, acid-fast rods were not found using Ziehl-Neelsen´s (ZN) stain. Additional findings besides the neoplasm included a mild to moderate, lymphoplasmacytic portal inflammation in liver and mucosal inflammation in the small intestine, which also encompassed heterophils in the liver. Additionally, the liver showed a mild fibrosis of portal areas adjacent to the tumour invasion.

Furthermore, selected sections of both cases were stained immunohistochemically using commercially available mono- or polyclonal antibodies against a broad panel of antigens (Table S1). Immunohistochemistry was performed using the avidin-biotin-peroxidase complex method (Vector Laboratories, Burlingame, USA) with 3, 3′-diaminobenzidine-tetrahydrochloride (DAB) as chromogen [38]. Positive controls were taken from archival blocks of duck organs and tissues originating from necropsy cases (Additional file 1; Additional file 2). Sections from both cases and archived tissue were incubated with a species-equivalent non-immune serum substituting the primary antibody and serving as negative controls.

The ectodermal compound of the teratoma showed expression of various cytokeratins (CKs; Additional file 1) labelled with the antibodies CK AE1 (specific for high molecular CKs 10, 14, 15, 16, 19)/AE3 (specific for high molecular CKs 1, 2, 3, 4, 5, 6 and low molecular CKs 7 and 8), HMW CKs (CK 1, 5, 10, 14), CK MNF 116 (specific for CKs 5, 6, 8, 17 and 19) as well as CK14 and CK20 in squamous epithelial cells. Single and bundled fibers of nerve tissue revealed immunoreactivity for pan-neurofilament (NF) interpreted as axonal structures (Fig. 1d). Furthermore, in one area of loosely arranged cells a weak neuron-specific enolase (NSE) expression was observed. Additionally, in a honeycomb-like tissue structure, anti-oligodendrocyte transcription factor (Olig2) labelled several round oligodendrocyte-like cells (Fig. 1e). Occasionally, clusters of glial fibrillary acidic protein (GFAP) expressing cells with extensive cytoplasmic processes resembling astrocytes were present (Fig. 1f). Concerning mesodermal tissue components, CD3 positive T-lymphocytes were randomly distributed and also found in aggregates (Fig. 1g). Muscle fibres multifocally showed an intense desmin (Fig. 1h) and smooth muscle actin (SMA) immunoreactivity. Vascular endothelial cells expressed factor VIII-related antigen. Sox2, which is a marker for the regulation of stem cell proliferation and differentiation [39], showed multifocal reactivity in cuboidal to columnar cells in tubular structures (Fig. 1i). The presence of neuroendocrine tissue was proofed by the weak positive signal for chromogranin A in cells around adipose tissue.

Ectodermal structures of the teratocarinoma expressed various CKs labelled with the antibodies AE1/AE3, HMW CKs and CK20 (Fig. 2f) in epithelial cells, partly forming keratin pearls. Weakly CK14 positive epithelial cells were also present (not shown). In contrast to the teratoma, ectodermal components of the teratocarcinoma did not show immunoreactivity for the pancytokeratin marker MNF116. Desmin (Fig. 2g) and smooth muscle actin (SMA, Fig. 2h) positive fibres were frequently present between tubular structures and around vessels. Additionally, weak factor VIII-related antigen positive vascular endothelial cells were observed. CD3 positive T-lymphocytes were found in a similar pattern as in the teratoma (Fig. 2i). GFAP expression was observed in clusters of cells between adenoid epithelial structures (Fig. 2j). Cuboidal to columnar cells in adenoid structures showed multifocal Sox2 immunoreactivity (Fig. 2k). In contrast to the teratoma, the remaining antibodies for labelling of nervous tissue (NF, NSE, Olig2) and neuroendocrine tissue (chromogranin A) did not show reactivity in the teratocarcinoma. Mesenchymal tissue in the teratoma and in the teratocarcinoma did not show vimentin reactivity, but mesenchymal tissue from a control duck show gradually variable immunolabelling. In addition, antibodies specific for CD79a (B-lymphocytes), CK5/6, CK7, CK10, synaptophysin (neuroendocrine cells) and CD31 (endothelial cells) did not show immunoreactivity in duck control tissue (Additional file 1).

## Discussion

The composition of tissues from all germinal layers in the teratoma of case 1 resembles the morphologic architecture of teratomas in mammals [3]. The current teratoma presents a neoplasm of unknown primordial basis regarding the organ system. Randomly sprinkled primordial pluripotent cells or coelomic organs represent possible origins.

The neoplasm of case 2 included tissues from all three primordial, germinal layers with a malignant transformation of the epithelial cells accompanied by fibrous tissue causing *lymphangiosis carcinomatosa* resulting in the diagnosis of a tridermic teratocarcinoma. The organ of neoplastic origin remains undetermined, although the ovaries represent a potential candidate. The inflammatory changes and the fibrosis could present secondary changes due to growth of the mass. Immunolabelling of cytokeratins and Sox2 identified ectodermal as well as endodermal cell populations as malignant cell populations in this teratocarcinoma. Sox2 expression has been described in mammals in odontogenic epithelium [40], cuboidal to columnar cells of salivary excretory ducts [41] and salivary glands [42]. In a case of a coccygeal teratoma in a cat, Sox2 was expressed in a transition stage from immature to mature non-myelinating Schwann cells, in immature, odontogenic epithelial cells and in immature, endodermal cells [9]. The morphology of the cuboidal to columnar cells in adenoid structures of the teratoma and the teratocarcinoma implies the presence of endodermal cells.

Regarding CKs in both tumours, the teratoma showed a broad expression for different CKs (CK 1,2,3,4,8,14,15,16,17,19,20), while the CK-labelling of the epithelial cells in the teratocarcinoma showed no immunoreactivity to MNF116, suggesting a slightly reduced expression of CKs (CK 1,2,3,4,8,14,15,16,20). This expression pattern may underline the lower grade of cellular differentiation in the teratocarcinoma, but could also be related to a different tumour composition and therefore reflecting the heterogeneity of teratomas and teratocarcinomas. Unfortunately, the expression of CKs in different avian epithelial tissue is not completely solved yet, but a similar expression of human and avian CKs is suspected [43, 44].

Mesenchymal components of both tumours did not show vimentin reactivity. In mesenchymal tissue from a control duck gradually variable immunolabelling of different mesenchymal cells was observed, so expression of vimentin may vary and could be below the detection level in the tumour tissue.

The presence of lymphocytes in teratomas and in teratocarcinomas of ducks has not been reported yet. In a human uterine teratoma, marked lymphoid infiltration has been interpreted as immunological reaction to the tumour cells [45]. In the current cases, the lymphocytes can either originate from the neoplasm itself (haematopoietically active bone marrow), but more likely from reactive infiltrates in case of clustered or random distribution within the neoplasm.

In addition, different components of nervous tissue were evident in both neoplasms, which was confirmed by immunohistochemistry. The teratoma contained NF-expressing axonal structures, Olig2-positive oligodendrocyte-like cells, GFAP-expressing astrocytic cells and isolated NSE-expressing cells, whereas only GFAP-expressing cells were detected in the teratocarcinoma. In a previously published case of a teratoma in a duck, immunohistochemistry revealed GFAP and NSE reactive tissue components [27]. Nevertheless, NF positive axons and Olig2 positive oligodendrocytes have not been reported in teratomas in ducks, yet. However, it has been described that neurons and oligodendrocytes were histologically noticeable in a teratoma in a duck [10]. GFAP positive astrocytes in avian teratocarcinomas have previously only been detected in an emu [35]. Thus, in case of indistinct nervous components, immunohistochemistry can provide additional valuable information about the composition of the neoplasm. The remaining antibodies underline the composition of teratomas and teratocarcinomas deriving from cell types of all primordial, germinal layers. Regarding immunohistochemical reactivity, the teratocarcinoma more often showed unspecific or lack of immunohistochemical reactivity compared to the teratoma implying a lower grade of differentiation particularly regarding tubular structures in the teratocarcinoma.

Besides teratomas and teratocarcinomas, other benign and malignant neoplasms originating from various epithelial, mesenchymal and neuroectodermal tissues have to be considered as potential differential diagnoses. In humans, the very rare condition of adenocarcinomas of the intestinal type in mature cystic teratoma of the ovary is described [46]. Malignant transformation of one of the components can occur towards squamous cell carcinomas [80%] or to adenocarcinomas [46], thus affecting ectodermal components. Moreover, adenocarcinomas of the female reproductive tract, in particular ovary or oviduct, should be considered as differential diagnosis. These can be accompanied by metastases with marked anaplasia. Rare squamous differentiation or osseus metaplasia may be confused with teratomas or teratocarinomas [47, 48]. Furthermore, on gross examination, granulomatous inflammations due to fungal or mycobacterial infections should be kept in mind. The cause of the granulomatous inflammation in case 2 remains undetermined.

In conclusion, although rare, teratomas and teratocarcinomas should be considered as differential diagnoses in ducks in case of coelomic masses. In most cases, teratomas and teratocarcinomas can be diagnosed by histological examination only. However, for the identification of particular tissue elements, immunohistochemical examination represents an additional, valuable tool. In the presented cases, the presence of nervous tissue and lymphocytes became obvious after the application of appropriate immunohistochemical markers.

Furthermore, immunohistochemical reactivity on duck tissue has been examined for a large spectrum of antibodies which could be helpful for the examination of duck tissue in the future.

## Electronic supplementary material

Below is the link to the electronic supplementary material.

Supplementary Material 1

Supplementary Material 2

Supplementary Material 3

## Data Availability

Data sharing is not applicable to this article as no datasets were generated or analysed during the current study.
