# Supplementary material for: Comparative immunohistochemical characterisation of a teratoma in a domestic duck (Anas platyrhynchos) and a teratocarcinoma in a muscovy duck (Cairina moschata)
Source: Acta Vet Scand. 2025 Apr 11;67:19. doi: 10.1186/s13028-025-00791-z (PMC11987307; doi:10.1186/s13028-025-00791-z)
Supplement: Supplementary file 1 — Supplementary Material 1 [file 13028_2025_791_MOESM1_ESM.docx]

**Table 1.** Antibodies used in immunohistochemistry and reactivity in the teratoma and teratocarcinoma as well as in control tissues of a duck

| *1^st^ antibody* | *Type* | *Dilution* | *Pretreatment* | *Supplier* | *2^nd^ antibody* | *Reactivity in the teratoma* | *Reactivity in the teratocarcinoma* | *Positive control tissues of a duck* |  |
| --- | --- | --- | --- | --- | --- | --- | --- | --- | --- |
| **Neuro-Ectodermal tissue** | | | | | | | | various epithelial tissues including skin with adnexal epithelial structures, cutaneous mucus membrane, intestine, kidney; brain; pancreas | |
| Pan-Cytokeratin [clone AE1/AE3, M3515], | Mouse monoclona | 1:500 | Microwave, citrate buffer | Dako, Hamburg, Germany | Goat anti mouse [GAM, biologo, BA-9200] | + (c) | + (c) | + (c) |  |
| Pan-Cytokeratin [clone MNF 116] |  | 1:100 |  |  |  | +(c) | - | + (c) |  |
| High-Molecular- Weight- Cytokeratin [HMW-Cytokeratin, clone 34βE12 (CK 1, 5, 10, 14)], |  | 1:500 |  |  |  | + (c) | + (c) | + (c) |  |
| Cytokeratin 5/6 (CK 5/6, clone D5/16B4) |  | 1:100 |  |  |  | - | - | - |  |
| Cytokeratin 7 (CK7, clone DE K10) |  | 1:50 |  |  |  | - | - | - |  |
| Cytokeratin 10 [CK10, clone DE-K10] |  | 1:500 |  |  |  | - | - | - |  |
| Cytokeratin 20 [CK20, clone Ks20.8] |  | 1:500 |  |  |  | + (c) | + (c) | + (c) |  |
| Cytokeratin 14 [CK14, PA5-16722] | Rabbit polyclonal | 1:500 | Microwave, citrate buffer | Thermo Fisher Scientific, Waltham, USA | Goat-anti- rabbit [GAR, 1:200, biologo, BA-1000] | + (c) | + (c) | + (c) |  |
| Glial fibrillary acidic protein [GFAP, clone 6F2] | Rabbit polyclonal | 1:1000 | None | Dako, Hamburg, Germany | Goat-anti- rabbit [GAR, 1:200, biologo, BA-1000] | + (c) | + (c) | + (c) |  |
| Pan-Neurofilament [NF, clone 2F11] | Mouse monoclonal | 1:100 | [Microwave, citrate buffer] |  | Goat anti mouse  [GAM, biologo, BA-9200] | + (c, n) | - | + (c, n) |  |
| Neuron-specific Enolase [NSE, clone BBS/NC/VI-H14**]** | Mouse monoclonal | 1:100 | None | Agilent Technologies, California, USA | Goat anti mouse [GAM, biologo, BA-9200] | + (c, n) | - | + (c, n) |  |
| Anti-Oligodendrocytes Antibody [Olig2, clone NS-1] | Mouse monoclonal | 1:500 | [Microwave, citrate buffer] | Sigma-Aldrich Chemie GmbH, Taufkirchen, Germany | Goat anti mouse [GAM, biologo, BA-9200] | + (n) | - | + (n) |  |
| Chromogranin A [DAK-A3] | Mouse monoclonal | 1:1000 | Microwave, citrate buffer | Dako, Hamburg, Germany | Goat anti mouse [GAM, biologo, BA-9200] | + (c) | - | + (c) |  |
| Synaptophysin [clone DAK-SYNAP] | Mouse monoclonal | 1:600 | [Microwave, citrate buffer] | Agilent Dako, Santa Clara, USA | Goat anti mouse [GAM, biologo, BA-9200] | - | - | - |  |
| **Mesodermal tissue** | | | | | | | | heart, spleen, kidney |  |
| CD3 T-lymphocytes [DAKO A0452] | Rabbit polyclonal | 1:200 | [Microwave, citrate buffer] | Dako, Hamburg, Germany | Goat-anti- rabbit [GAR, 1:200, biologo, BA-1000] | + (c) | + (c) | + (c) |  |
| CD79a [clone HM57] | Mouse monoclonal | 1:5000 | [Microwave, citrate buffer] |  | Goat anti mouse [GAM, biologo, BA-9200] | - | - | - |  |
| CD31 [PECAM1] [C-term] | Rabbit polyclonal | 1:100 | [Microwave, citrate buffer] | Origene, Rockville, USA | Goat-anti- rabbit [GAR, 1:200, biologo, BA-1000] | - | - | - |  |
| Vimentin [clone V9] | Mouse monoclonal | 1:100 | None | Agilent Dako, Santa Clara, USA | Goat anti mouse [GAM, biologo, BA-9200] | - | - | + |  |
| Desmin [clone D33] | Mouse monoclonal | 1:100 | None | Dako, Hamburg, Germany | Goat anti mouse [GAM, biologo, BA-9200] | + (c) | + (c) | + (c) |  |
| Smooth Muscle Actin [SMA, clone 1A4] | Mouse monoclonal | 1:200 | None |  | Goat anti mouse  [GAM, biologo, BA-9200] | + (c) | + (c) | + (c) |  |
| Factor VIII related antigen/von Willebrand Factor [A0082 | Rabbit polyclonal | 1:500 | Microwave, citrate buffer |  | Goat-anti- rabbit [GAR, 1:200, biologo, BA-1000] | + (c) | + (c) | + (c) |  |
| **Miscellaneous antibodies** | | | | | | | | Brain |  |
| Sox2 | Rabbit monoclonal | 1:50 | Microwave, citrate buffer | Cell Signaling Technology Inc., Danvers | Goat-anti- rabbit [GAR, 1:200, biologo, BA-1000] | + (c, n) | + (c, n) | + (c, n) |  |

Localisation of the immunohistochemical signal: c = cytoplasmic; n = nuclear; immunoreactivity: + = positive; - = negative;
