# Supplementary figures and images for: Comparative immunohistochemical characterisation of a teratoma in a domestic duck (Anas platyrhynchos) and a teratocarcinoma in a muscovy duck (Cairina moschata)
Source: Acta Vet Scand. 2025 Apr 11;67:19. doi: 10.1186/s13028-025-00791-z (PMC11987307; doi:10.1186/s13028-025-00791-z)

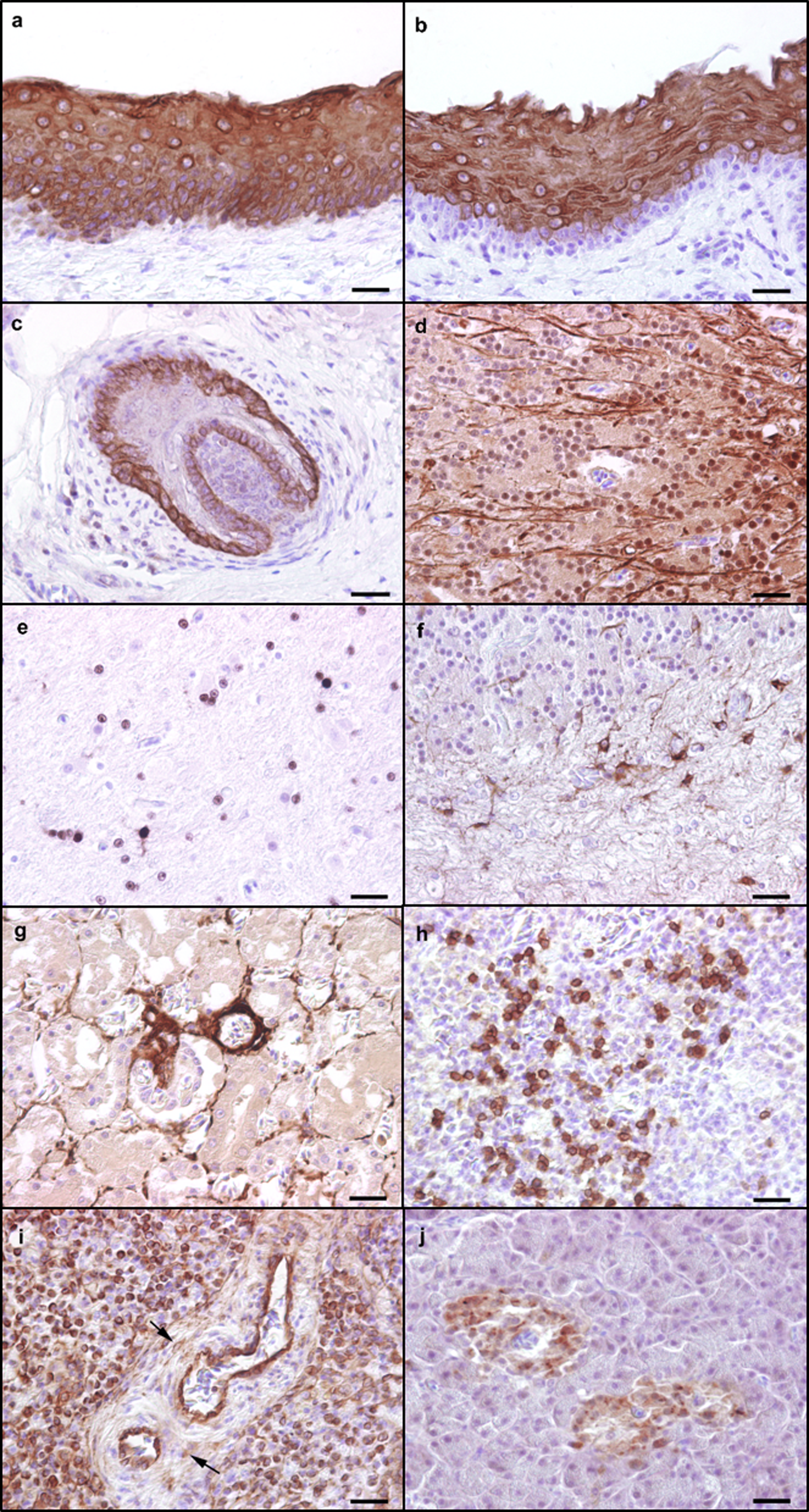

Supplement: Supplementary file 2 — Supplementary Material 2 [file 13028_2025_791_MOESM2_ESM.tif]
