# Supplementary material for: Comparative immunohistochemical characterisation of a teratoma in a domestic duck (Anas platyrhynchos) and a teratocarcinoma in a muscovy duck (Cairina moschata)
Source: Acta Vet Scand. 2025 Apr 11;67:19. doi: 10.1186/s13028-025-00791-z (PMC11987307; doi:10.1186/s13028-025-00791-z)
Supplement: Supplementary file 3 — Supplementary Material 3 [file 13028_2025_791_MOESM3_ESM.docx]

**Additional file 1 (Suppl_Fig. 1).** Examples of positive immunostaining in duck control tissues.

a) Pan CK AE1/3 staining of all layers of a cutaneous mucus membrane;

b) Selective immunolabelling of the suprabasal layers of a cutaneous mucous membrane with Pan CK MNF116;

c) Feather bulb with CK14 expression in basal cells;

d) Extensive expression of neurofilament in neuronal processes of the cerebellum;

e) Olig2 expression in oligodendrocytes of the brain stem;

f) GFAP expression in astrocytes of the cerebellum;

g) Smooth muscle actin expression in vascular wall cells of a small arteriole, the glomerular vascular tuft and interstitial cells of the kidney;

h) CD3-positive T lymphocytes in the spleen;

i) Vimentin expression of mesenchymal cells in the spleen with intense labelling of lymphoid and vascular endothelial cells and very weak staining of vascular wall cells;

j) Chromogranin A expression in cells of the endocrine pancreas;

bar: 50 µm
